# MRI Brain Lesions in Eclampsia: A Series of 50 Cases Admitted to HDU of a Tertiary Care Hospital 

**Published:** 2018-03

**Authors:** Krishna Dahiya, Seema Rohilla, Kriti Agarwal, Mahesh Rathod, Archit Dahiya

**Affiliations:** 1 Department of Obstetrics and Gynecology, PGIMS, Rohtak, India; 2 Department of Radiodiagnosis, PGIMS, Rohtak, India; 3 Department of Medicine, PGIMS, Rohtak, India

**Keywords:** Eclampsia, MRI, Neurological

## Abstract

**Objective:** To correlate the Magnetic Resonance Imaging findings and clinical presentation in patients of eclampsia.

**Materials and methods:** This one year prospective study was conducted in the HDU of Department of Obstetrics and Gynecology, PT.B.D.Sharma, PGIMS, Rohtak .A total of 50 women with eclampsia (both antepartum and postpartum) were divided into two groups: a) study group patients with abnormal MRI b) control group: b) control group: patients with normal MRI.Comparison was done using chi-square test and unpaired student ‘t’ test.

**Results:** MRI revealed abnormal findings in 24% of women, commonest diagnosis being CVT without infarct (10%) followed by infarct (8%), PRES (4%) and HLE (2%).Totally 66% (n = 33) of the women presented with postpartum eclampsia while 34% (n = 17) had antepartum eclampsia.96%(n = 48) were unbooked cases. Unconsciousness, altered sensorium, headache, blurring of vision, seizures, GCS < 3 correlated well with MRI findings (p = 0.000, p = 0.027, p = 0.001, p = 0.007, p = 0.005, p = 0.000 respectively) whereas fundoscopic changes did not (p = 0.520). The mean uric acid and serum creatinine levels was higher (0.41 ± 0.11 mmol/ L vs 0.26 ± 0.10 mmol/ L and 80 ± 18 µmol/ L vs 71 ± 9 µmol/ L) in the study group and this was statistically significant (p = 0.003, p = 0.04 respectively).There was no statistically significant difference between blood pressure values of cases with or without MR imaging evidence of brain lesions. There was no maternal mortality among 50 cases. The sensitivity, specificity, positive predictive value and negative predictive value of neurological findings for abnormal MRI in patients with eclampsia was found to be 91.7%, 73.7%, 52.4%, 96.6% respectively.

**Conclusion:** Unconsciousness, altered sensorium, headache, blurring of vision,seizures, GCS < 3, elevated uric acid and serum creatinine levels in the follow-up of pregnant patients with preeclampsia/eclampsia should be a warning for possible brain lesions whereas booking status, mean BP, fundoscopy, platelet, hemoglobin, liver enzymes were not significantly associated with positive MRI findings in patients of eclampsia.

## Introduction

Eclampsia is an acute neurological complication of preeclampsia characterized by seizures and/or consciousness disorders which cannot be related to another neurological disease. The incidence of eclampsia is around 1 in 2000 deliveries in developed countries and as high as around 1 in 100 to 1 in 1700 in developing countries (1). Eclampsia is associated with an increased risk of maternal death in developed countries (0%-1.8%), but the mortality rate is as high as 15% in developing countries (2). Among the morbidity factors, neurological involvement plays a major role. This neurological disease was first described in 1881 by the discovery of cerebral hemorrhage in eclampsia (3). Recent studies using computed tomography (CT) and magnetic resonance imaging (MRI) help to better understand brain lesions that occur during an eclamptic fit. These lesions include intracerebral hemorrhage, cerebral ischemia, and cerebral edema (4, 5). The objectives of the present study were to correlate the Magnetic Resonance Imaging findings and clinical presentation in patients of eclampsia.

## Materials and methods

This one year prospective study was conducted in the HDU of Department of Obstetrics and Gynecology, PT.B.D.Sharma, PGIMS, Rohtak .A total of 50 women with eclampsia (both antepartum and postpartum) were divided into two groups: a) study group :patients with abnormal MRI b) control group :patients with normal MRI. Women who were known case of hypertension, epilepsy, seizures due to metabolic disturbances, space occupying lesions or intracerebral infections were excluded from study. Detailed history was elicited and all patients were subjected to investigations such as hemoglobin, 24 hour urine protein, renal function tests, liver function tests, absolute platelet count and fundoscopy.

***Statistical analysis:*** The categorical data was expressed as rates, ratios and proportions and continuous data was expressed as mean ± standard deviation (SD). The comparison was done using chi-square test and unpaired student‘t’ test. Sensitivity, specificity, positive predictive value and negative predictive value were calculated to find the accuracy of neurological presentation in determining the MRI diagnosis. A probability value (p value) of less than 0.050 was considered as statistically significant.

## Results

During one year period, 50 eclamptic women underwent MR Imaging. 24% (n = 12) patients had findings on MRI. Accordingly, the study was divided into two groups, study group in which patients had findings on MRI and compared with control group in which patients had no findings on MRI. 

50% (n = 25) presented with age between 22 to 25 years of which 6 patients showed findings on MRI. The mean age of the study population was 22.61 ± 2.72 years. Age wise distribution of patients in case and control group was statistically not significant (p value: 0.136) (Table 1).

**Table 1:** Age distribution

**Age ** **groups ** **(years)**	**Study ** **group (MRI ** **findings ** **present)**	**Control ** **Group (MRI ** **findings ** **absent)**	**Total ** **patients**
21 or less	3	17	20
22 to 25	6	19	25
26 to 28	2	2	4
> 28	1	0	1
Total	12(24%)	38(76%)	50(100%)

p value : 0.136 (not significant)

96% (n = 48) were unbooked, however booking status in both groups was statistically non significant (P value: 0.380). In this study, 86% (n = 43) belonged to rural background and 14% (n = 7) to urban area. Geographic distribution in both groups was statistically significant (p value-0.027) (Table 2). 

**Table 2:** Geographic distribution (n = 50)

**Geographic ** **distribution**	**Study ** **group**	**Control ** **Group**	**Total ** **patients**
Urban	4	3	7
Rural	8	35	43
Total	12	38	50

p value : 0.027 (significant)

Totally 58% (n = 29) were primi parous and 42% (n = 21) were multi parous. The mean gestational age in women with antepartum eclampsia was 35.56 ± 3.17 weeks and in post partum eclampsia, the mean day of presentation was 3.82 ± 1.93 days. Totally 66% (n = 33) of the women presented with post partum eclampsia while 34% (n = 17) had ante-partum eclampsia (Table 3). Unconsciousness, altered sensorium, fits, headache, blurring of vision was significantly higher in the study group where as mean BP had no significant difference in the two groups. 

**Table 3:** Time of Presentation

**Presentation**	**Study ** **group**	**Control ** **Group**	**Total**
Antepartum	4	13	17(34%)
Postpartum	8	25	33(66%)
Total	12	38	50(100%)

p value: 0.202 (not significant)

44% (n = 22) presented with more than one clinical presentation (Table 4). 

**Table 4:** Clinical presentation

**Presentation**	**Study group ** **(n = 12)**	**Control group ** **(n = 38)**	**p value**
Unconscious	11	6	0.000
Altered sensorium	4	3	0.027
3 or less fits	7	35	0.005
4 or more fits	5	3	0.005
Frothing	1	5	0.654
Incontinence	1	2	0.696
Headache	10	11	0.001
Blurring of vision	6	5	0.007
Vomiting	1	9	0.246
Mean SBP	156.46 ± 13.03 mm of hg,	148.28 ± 13.56	0.404
Mean DBP	101.62 ± 6.94 mm of Hg	96.42 ± 7.12 mm of Hg	0.404

GCS < 3 was significantly higher in the study group. (p value 0.000) (Table 5). 

**Table 5:** Glassgow coma scale

**GCS**	**Study group**	**Control group**	**p value**
< 3	5	1	0.000
3-10	5	27	0.064
10-15	2	10	0.495
Total	12	38	-

Fundoscopic changes in both study and control group was statistically non-significant (p value: 0.520) (Table 6). The mean uric acid levels among study group was 0.41 ± 0.11 mmol/ L and in control group it was 0.26 ± 0.10 mmol/ L, which was found statistically significant (p value 0.003). Mean serum creatinine levels in study group was found to be 80 ± 18 µmol/ L and in control group it was found to be 71 ± 9 µmol/ L (p value 0.04) (Table 7).

**Table 6:** Fundoscopy

**Grade**	**Study group**	**Control group**
Normal	9	35
Grade 2	1	2
Grade 3	1	1
Grade 4	1	0
Total	12	38

Complications like status epilepticus, pulmonary oedema , ARF was seen in 4% (n = 2) of cases where as PPH, abruption, DIC, aspiration pneumonia were found in 2% (n = 1). 

**Table 7:** Laboratory investigations

**Clinical data ** **(normal range)**	**Study group ** **(n = 12)**	**Control group ** **(n = 38)**	**p value**
WBC count (4-10 ×10^9^/ L)	12.6 ± 5.8	9.6 ± 2.4	0.13
Platelets (150-450 ×10^9^/ L)	198 ± 102	232 ± 108	0.52
AST (9-30 U/ L)	32 ± 16	31 ± 14	0.92
ALT (7-52 U/ L)	38 ± 27	39 ± 28	0.95
Uric acid (0.11-0.38 mmol/ L)	0.41 ± 0.11	0.26 ± 0.10	0.003 (s)
Serum creatinine (71-115 µmol/ L)	80 ± 18	71 ± 9	0.04 (s)

Three cases of each HELLP and neurologic deficit were found. There was no maternal mortality among 50 cases (Table 8).

**Table 8:** Maternal outcome

**Complications **	**n (%)**
Status eclampticus	2 (4%)
PPH	1 (2%)
Abruption	1 (2%)
Pulmonary oedema	2 (4%)
ARF	2 (4%)
DIC	1 (2%)
Aspiration pneumonia	1 (2%)
HELLP	3 (6%)
Neurologic deficit	3 (6%)

On MRI, commonest diagnosis was CVT without `infarct (10%) followed by infarct (8%), PRES (4%) and HLE (2%) (Table 9). A group of 21 patients had neurological signs and symptoms of eclampsia, out of which 11 patients showed positive MRI findings and one showed negative MRI findings. 29 patients had no neurologic presentation, out of which one showed positive MRI findings and 28 showed negative MRI findings.

**Table 9:** Magnetic resonance imaging findings

	**n (%)**
CVT without infarct	5 (10.00)
Infarct	4 (8.00)
Posterior reversible encephalopathy syndrome	2 (4.00)
Hypertensive leucoencephalopathy	1 (2.00)
No abnormality detected	38 (76.00)
Total	50 (100.00)

The sensitivity, specificity, positive predictive value and negative predictive value were found to be 91.7%, 73.7%, 52.4%, 96.6% (Table 10).

**Table 10:** Accuracy of neurological signs and symptoms in determining the MRI diagnosis

**Neurological signs ** **and symptoms**	**MRI findings**		**Total**
	**Positive**	**Negative**	
Present	11	10	21
Absent	1	28	29
Total	12	38	50

Sensitivity = 91.7%, Specificity = 73.7%, PPV = 52.4%, NPV = 96.6%

## Discussion

Brain lesions detected in eclampsia might be related to a disturbance of the cerebral autoregulation mechanism and impairment of endothelial function (6- 8).The cerebral autoregulation mechanism, consisting of myogenic and neurogenic components, maintains stable blood perfusion in normal individuals .(6) Effective functioning of neurogenic mechanisms depends on sympathetic innervation. Direct toxic effects on endothelium or vessel distention, which depends on elevated blood pressure, decreases the effect of myogenic mechanisms (6). In these cases, neurogenic mechanisms take over the regulation of cerebral perfusion; this way, posterior circulation areas, which are relatively sparsely innervated by sympathetic nerves, become more sensitive to blood pressure elevations. In cases with hypertension, serum extravasation occurs when the elevation in blood pressure passes beyond the autoregulation capacity of brain blood vessels. Brain lesions are more commonly demonstrated in posterior areas in these cases (9). In cases without hypertension, direct endothelial cell dysfunction, which increases blood-brain barrier permeability, is thought to be responsible for the pathogenesis (6, 7).

 In this study, various clinical and laboratory parameters in eclampsia cases with and without positive brain MR imaging findings were compared. 50% (n = 25) were aged between 22 to 25 years and 40% of the women were aged ≤ 21 years. A study by Jindal MA reported 30 patients belonged to with age between 20 to 25 years (60%) which was comparable with the present study (10).The mean age in this study was 22.61 ± 2.72 years. A similar study by Kokila MS et al reported the mean maternal age as 23.89 years (range 18-30 years) (11). Totally 66% (n = 33) women had postpartum eclampsia while 34% (n = 17) had antepartum eclampsia. These results were also similar to the epidemiological and interventional studies conducted by Majiko et al in the developing countries like Zimbabwe (12). In a series of eight cases seizure onset was more in antepartum (5 cases) than postpartum (3 cases) (13). In our study, 58% (n = 29) were primiparous and 42% (n = 21) were multi parous. These findings were consistent with a study by Jindal MA et al who reported that most of their patients as primi parous (56%) (10).

Unconsciousness, altered sensorium, headache, blurring of vision, seizures, GCS < 3 correlated well with MRI findings (p = 0.000, p = 0.027, p = 0.001, p = 0.007, p = 0.005, p = 0.000 respectively) where as fundoscopic changes did not (p = 0.520). A study in Bangladesh showed that there were statistically significant difference between the two groups regarding headache, visual disturbance, hypereflexia & depression of consciousness similar to our findings where as aphasia & hemiplegia did not contribute significantly (14). A Taiwanese study also showed comparable radiological findings in patients of eclampsia with headache and blurring of vision (15). However study in Turkey showed that headache was not significantly associated with positive MRI findings (9).

The mean uric acid and serum creatinine levels was higher (0.41 ± 0.11 mmol/ L vs 0.26 ± 0.10 mmol/ L and 80 ± 18 µmol/ L vs. 71 ± 9 µmol/ L) in the study group and this was statistically significant (p = 0.003, p = 0.04 respectively) . Schwartz et al.^6^ demonstrated that brain lesions detected in MR examination are associated with endothelial damage indicators, not hypertension .According to them, abnormal red cell morphology and LDH levels were found to be significantly higher in those patients of eclampsia with abnormal MRI findings and blood pressures were not significantly different between groups at any time similar to our study. Similarly, in a Turkish study biochemical data like lactate dehydrogenase (LDH), uric acid, and creatinine levels were significantly higher in patients with positive MR findings than those without MR findings (9). The cause of endothelial injury in preeclampsia/eclampsia cases has not yet been demonstrated, but circulating endothelial toxins or endothelium antibodies are thought to be responsible (6). In previous studies, it has been demonstrated that in preeclampsia/eclampsia patients, high LDH levels occur before lesions appear in brain MR examination, and this finding shows that high blood pressure does not lead to endothelial injury (6, 16). Renal function disorder secondary to renal endothelial injury results in uric acid and creatinine increase (9). In our study, there was no statistically significant difference between blood pressure values of cases with or without MR imaging evidence of brain lesions. But in cases of preeclampsia/ eclampsia, brain lesions might occur although blood pressure values are normal but still higher than a patient’s routine normal blood pressure (6). The distribution of brain lesions should also be evaluated in determining whether hypertension or endothelial injury is more responsible for the pathogenesis (9).

Complications like status epilepticus, pulmonary oedema and ARF, were seen in 4% (n = 2) each whereas PPH, abruption, DIC, aspiration pneumonia were found in 2% (n = 1) each. HELLP and neurologic deficit were found in 6% (n = 3) of cases. There was no maternal mortality among 50 cases. Complications like HELLP syndrome, renal failure and acute pulmonary edema were seen in 33.33% (n = 13), 18% (n = 7), 5% (n = 2) respectively and mortality rate was 10.2% (n = 4) in a study conducted at ICU in Cocody (17).

MRI revealed abnormal findings in 24% of women, commonest diagnosis being CVT without infarct (10%) followed by infarct (8%), PRES (4%) and HLE (2%). A study by Kokila MS reported 46.4% (n = 13) of women had other noneclamptic organic causes for postpartum seizures and 28.6% (n = 8) of postpartum seizures were due to CVT (11). In a French study of eclamptic women with persistent neurological symptoms, all (n = 19) had abnormal neuroradiological findings on CT and MRI. Latter was performed in four cases and showed three cases of cerebral venous thrombosis and two cases of intracerebral haemorrhage (5).

Among the 54 patients admitted to the ICU at Cocody for eclampsia, brain lesions were found in 19 patients (48.7%) on CT imaging. These were ischemia: 10 cases (25.6%), bruising intraparenchymal: 3 cases (7.7%), and subarachnoid hemorrhage: 1 case (2.5%), cerebral edema: 9 cases (23%). The CT scan came back to normal 51% (n = 20) eclamptic patients (17).

Another study in Bangladesh, out of 35 patients, 85.72% had changes in brain on CT scan(14).Among them, 45.72% patients had cerebral oedema, 37.14% had cerebral infarct and 2.86% patients had intracerebral haemorrhage (14). The incidence of appearance of brain lesions in radiologic study is greatly affected by the temporal relationship of the scan to the development of seizure (15). In fact, in the French study, the MRI exhibited some edematous brain lesions in eclamptic patients who had a normal CT scan (5).

The sensitivity, specificity, positive predictive value and negative predictive value of neurological findings for abnormal MRI in patients with eclampsia was found to be 91.7%, 73.7%, 52.4%, 96.6% respectively. A prospective observational study was conducted by Jindal MA to compare CT and MRI findings of eclampsia patients with respect to neurological signs and symptoms reported that MRI was found to be co-relating more than CT with the neurological presentation and had 90% sensitivity and 100% sensitivity (10). In contrast, a study by Kokila MS et al concluded that, despite many abnormalities seen on imaging studies, some are incidental and transient and indicated in atypical and fatal cases (11).

## Conclusion

Unconsciousness, altered sensorium, headache, blurring of vision,seizures, GCS < 3, elevated uric acid and serum creatinine levels in the follow-up of pregnant patients with preeclampsia/eclampsia should be a warning for possible brain lesions where as booking status, mean BP, fundoscopy, platelet, hb, liver enzymes were not significantly associated with positive MRI findings in patients of eclampsia.
